# Parrotfish predation drives distinct microbial communities in reef-building corals

**DOI:** 10.1186/s42523-020-0024-0

**Published:** 2020-02-10

**Authors:** Leïla Ezzat, Thomas Lamy, Rebecca L. Maher, Katrina S. Munsterman, Kaitlyn M. Landfield, Emily R. Schmeltzer, Cody S. Clements, Rebecca L. Vega Thurber, Deron E. Burkepile

**Affiliations:** 1 Department of Ecology, Evolution and Marine Biology, University of California Santa Barbara, Santa Barbara, CA USA; 2 Department of Microbiology, Oregon State University, Corvallis, OR USA; 3 School of Biological Sciences and Aquatic Chemical Ecology Center, Georgia Institute of Technology, Atlanta, GA USA; 4 Marine Science Institute, University of California Santa Barbara, Santa Barbara, CA USA

**Keywords:** Parrotfish, Coral reefs, 16S rRNA gene, Corallivory, Bacteria, Corals, Vector, Transmission, Microbiomes

## Abstract

**Background:**

Coral-associated microbial communities are sensitive to multiple environmental and biotic stressors that can lead to dysbiosis and mortality. Although the processes contributing to these microbial shifts remain inadequately understood, a number of potential mechanisms have been identified. For example, predation by various corallivore species, including ecologically-important taxa such as parrotfishes, may disrupt coral microbiomes via bite-induced transmission and/or enrichment of potentially opportunistic bacteria. Here, we used a combination of mesocosm experiments and field-based observations to investigate whether parrotfish corallivory can alter coral microbial assemblages directly and to identify the potentially relevant pathways (e.g. direct transmission) that may contribute to these changes.

**Results:**

Our mesocosm experiment demonstrated that predation by the parrotfish *Chlorurus spilurus* on *Porites lobata* corals resulted in a 2-4x increase in bacterial alpha diversity of the coral microbiome and a shift in bacterial community composition after 48 h. These changes corresponded with greater abundance of both potentially beneficial (i.e. *Oceanospirillum*) and opportunistic bacteria (i.e. Flammeovirgaceae, Rhodobacteraceae) in predated compared to mechanically wounded corals. Importantly, many of these taxa were detectable in *C. spilurus* mouths, but not in corals prior to predation. When we sampled bitten and unbitten corals in the field, corals bitten by parrotfishes exhibited 3x greater microbial richness and a shift in community composition towards greater abundance of both potential beneficial symbionts (i.e. *Ruegeria*) and bacterial opportunists (i.e. Rhodospiralles, *Glaciecola*). Moreover, we observed 4x greater community variability in naturally bitten vs. unbitten corals, a potential indicator of dysbiosis. Interestingly, some of the microbial taxa detected in naturally bitten corals, but not unbitten colonies, were also detected in parrotfish mouths.

**Conclusions:**

Our findings suggest that parrotfish corallivory may represent an unrecognized route of bacterial transmission and/or enrichment of rare and distinct bacterial taxa, both of which could impact coral microbiomes and health. More broadly, we highlight how underappreciated pathways, such as corallivory, may contribute to dysbiosis within reef corals, which will be critical for understanding and predicting coral disease dynamics as reefs further degrade.

## Background

Reef-building corals host a wide range of microorganisms including endosymbiotic dinoflagellates (*Symbiodinaceae*), viruses, archaea, and bacteria that collectively comprise the coral holobiont [1]. The relationship between corals and these microbial associates allows reef corals to thrive in nutrient-poor waters and to support high biodiversity [2]. While the coral-dinoflagellate symbiosis is well documented, less is known about the roles of coral-associated bacterial communities in coral health and resilience [3–5]. However, an array of mutualistic benefits are suggested, encompassing vital functions such as coral nutrition and immunity that may further impact fundamental ecological processes within coral reefs [4, 6, 7].

Despite their beneficial role in host fitness, coral-associated bacteria are sensitive to numerous environmental and biotic stressors that may lead to microbial dysbiosis (i.e., a shift in either the mean composition or variability of the microbiome, including the loss of beneficial symbionts and/or increase of opportunists) [8, 9]. However, the processes that contribute to dysbiosis remain inadequately understood [8, 10–12]. For example, trophic interactions that are common within reef ecosystems, such predation on corals (i.e. corallivory), may favor the disruption of coral microbiomes and subsequently alter coral health [6, 8, 9, 13].

A variety of corallivores, including invertebrates such as fireworms [14], echinoderms [15], and gastropods [16, 17], may serve as reservoirs and/or vectors of opportunistic bacteria (i.e. typically non-pathogenic microorganisms that take advantage of their host under certain circumstances) or pathogens (i.e. microorganisms that cause infection) to corals. Corallivorous, polyp-feeding butterflyfishes may also spread microbes and parasites via their mouthparts [14–18]. However, the fact that butterflyfishes remove limited coral tissue without exposing the underlying skeleton may make them unlikely candidates to transmit microbes among individual corals [16]. In contrast, many parrotfishes scrape or excavate both live coral tissue and skeleton, while also ingesting detritus and turf algae from rocky surfaces [19]. This invasive feeding method and more varied diet make them likely candidates for disrupting coral microbiomes. Parrotfishes play a critical role in structuring the benthic communities of coral reefs and are generally considered to have a net positive effect on ecosystem functions, promoting coral dominance by removing competing macroalgae [19] and acting as important agents of reef bioerosion [20]. That said, parrotfish predation is a chronic stress that may cause significant harm to corals, especially when coupled with other environmental and/or biotic stressors. For example, recent findings suggest that corals exposed to both parrotfish predation and nutrient pollution experienced significantly greater mortality than when exposed to either stressor alone, which was attributed to increased bacterial opportunism [13]. Identifying the mechanisms and conditions in which parrotfishes can disrupt coral microbiomes will improve our ability to predict the microbial impacts associated with corallivory and their potential implications for coral health.

Here, we conducted a series of experiments in Mo’orea, French Polynesia, to assess the effects of parrotfish corallivory on coral microbiomes. Specifically, we tested whether parrotfish can facilitate the enrichment and/or transmission of microbes to corals. We focused on *Chlorurus spilurus*, a common parrotfish species known to prey on large colonies of *Porites* on Pacific reefs [21]. We first performed a controlled mesocosm experiment comparing the microbiomes of *Porites lobata* colonies that were either mechanically wounded or predated by *C. spilurus* parrotfish. Comparisons were based on samples collected immediately following predation or wounding (T_i_) and at 48 h (T_f_). To examine microbiome patterns in situ, we collected microbial samples from corals in the field that had either been naturally bitten by parrotfishes or appeared bite free (hereafter “unbitten”). Coral microbiomes from both the mesocosm experiment and field survey were also compared to microbiomes from parrotfish mouthparts to assess potential predation-mediated pathways (e.g. transmission) that may contribute to coral microbiome change. We hypothesized that predation by *C. spilurus* facilitates the enrichment and/or transmission of microbes to corals, resulting in the following impacts on the coral microbiome: (1) increases in alpha diversity, (2) differences in community composition (3) increases in community variability, and (4) increased abundance of microbial taxa typically found in the mouths of parrotfish but absent in healthy corals.

## Results

### Experimental overview

The impacts of parrotfish corallivory on coral microbiomes were assessed using a combination of manipulative experiments and field surveys on the north shore of Mo’orea, French Polynesia. First, to assess the ability of *C. spilurus* to feed on live *Porites lobata* corals, we conducted a survey in two back reef areas to quantify the number of *C. spilurus* bites found on live versus dead corals. A manipulative experiment was then performed at the UC Gump Marine station with seven *C. spilurus* initial phase individuals and ten colonies of *Porites lobata* (about 20 cm) that were previously collected on a nearby back reef. When at the station, sterile culture swabs were used to collect microbial samples of the mouths of each *C. spilurus* individual*.* Each *C. spilurus* was then coaxed into biting a single *Porites lobata* colony at two separate locations. The remaining three *P. lobata* colonies were artificially wounded at two locations using a sterilized bone cutter. Coral samples (mucus, tissue and part of the coral skeleton) were collected: i) immediately following parrotfish biting/mechanical wounding (Ti) and ii) at the end of the experiment (48 h, Tf). Coral samples and fish swabs were stored at − 80 °C prior to laboratory analyses.

To compare our experimental results with conditions in the field, we haphazardly collected *P. lobata* samples (mucus, tissue and part of the skeleton) that were either unbitten or naturally bitten (*n* = 10 per status) from a reef on Mo’orea’s north shore. Sterile swabs were also used to collect microbial samples from ten *C. spilurus* collected haphazardly from the same reef. Four 1 L-water samples were also collected and directly filtered on 0.2 um filters. When on the boat, coral tissues, swabs and filters were placed on ice and stored at − 80 °C immediately upon arrival at the marine station.

DNA extractions on all samples were performed using DNeasy PowerSoil Kit (Qiagen) and AccuStart II PCR ToughMix (Quanta BioSciences, Gaithersburg, Maryland, USA) was used to perform two-step Polymerase Chain Reaction (PCR) on the V4 hypervariable region of the 16S rRNA gene. Data processing and analyses of microbiome diversity, composition and stability metrics of corals, fish mouths and water were then performed using the Delbur workflow [22], QIIME2 [23] pipelines and R [24] for statistical analyses.

### Parrotfish feed on live corals in the field

Among the 23 individual fish that were followed, we recorded a total of 5451 bites on either live or dead corals (including rubble and pavement). Of these, 5400 (99%) were taken from dead corals and 51 (~ 1%) were taken from live corals, with the latter comprising mostly massive Porites colonies (49 bites, 96% of the bites on live coral were taken on massive Porites).

### Bacterial assemblages differed between parrotfish mouth, coral, and water samples

#### Mesocosm experiment

**Fig. 1:**
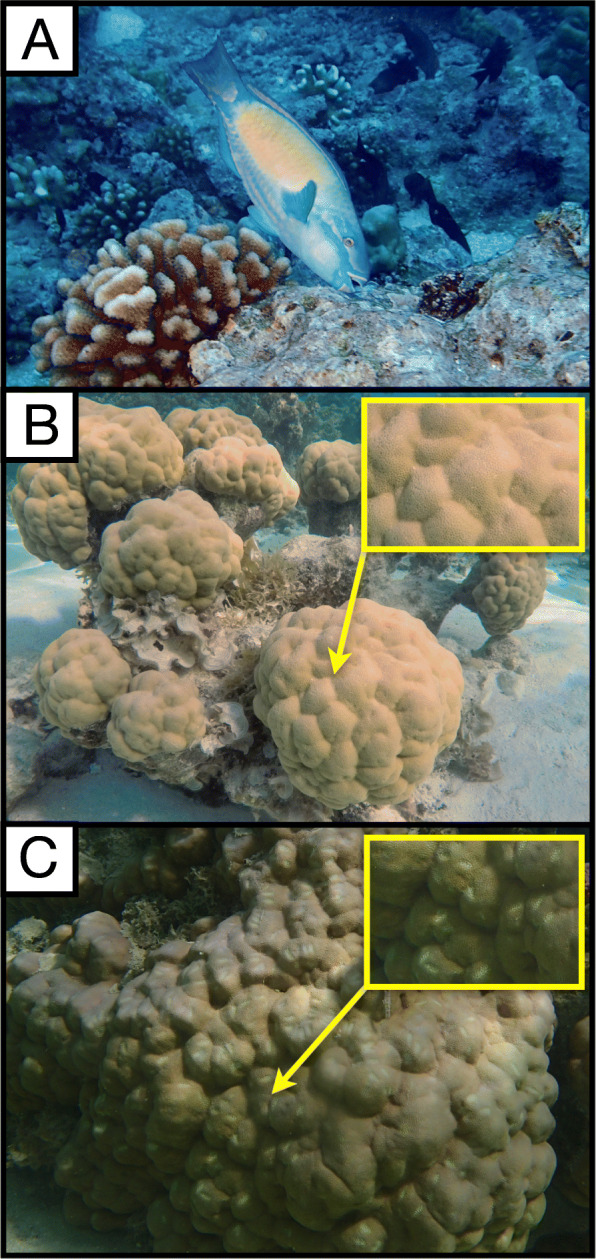
**a** Bullethead parrotfish *Chlorurus spilurus* (photo credit: Katrina Munsterman). **b** Unbitten colonies of *Porites lobata* (photo credit: Cody Clements) and **c** naturally bitten colonies by parrotfish as found in our study site in the back reef area of Mo’orea, French Polynesia (photo credit: Mallory Rice)

**Fig. 2:**
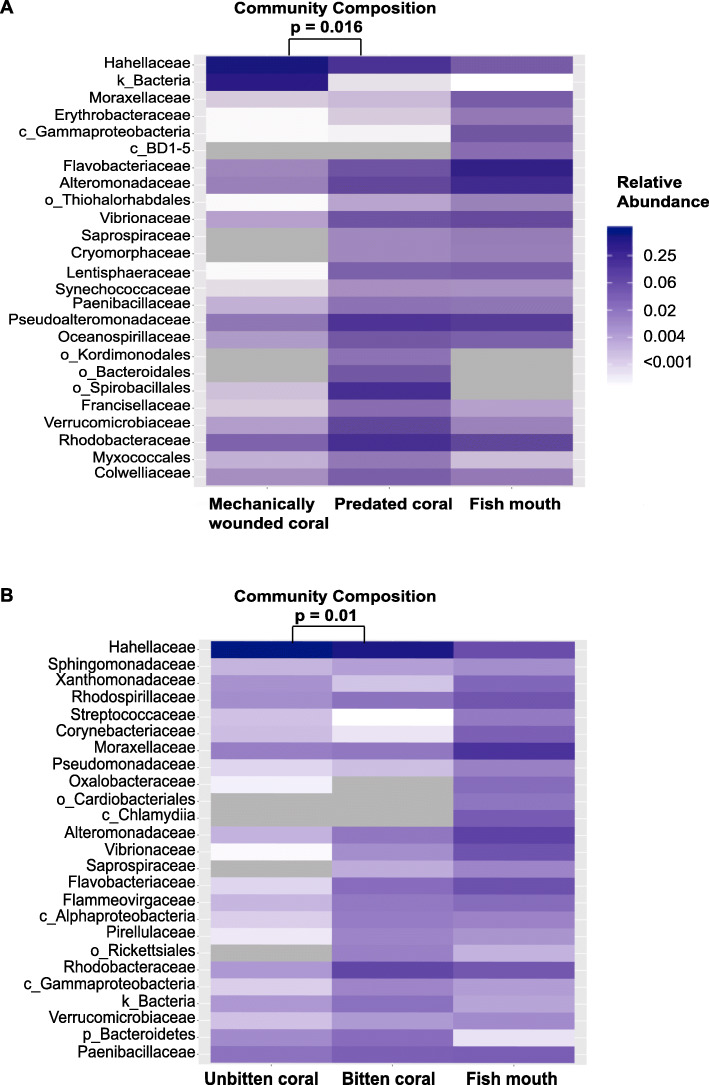
Heat maps displaying the relative abundance (expressed as proportion) of the 25 most abundant taxa grouped at the family level or to the closest taxonomic rank according to the sample type for (**a**) the mesocosm experiment (at T_f_) and (**b**) the field survey. *P*-values represent significant differences, based on pairwise comparisons using the pairwise.adonis function, in microbial community composition between **a** predated and mechanically wounded corals in the mesocosm experiment or **b** bitten and unbitten corals in the field

Parrotfish mouths showed distinct bacterial communities compared to mechanically wounded corals both at Ti (Additional file 1: Tables S1 and S2; pairwise Adonis; *p* = 0.02) and T_f_ (pairwise Adonis; *p* = 0.012) in the mesocosm experiment. Phylum level assignments in parrotfish mouths showed the dominance of by Proteobacteria and Bacteroidetes (Additional file 2: Figure S1). Among the 83 families characterized in parrotfish mouths, few were present at proportions greater than 1%. These included Flavobacteriaceae (23.1%), Alteromonadaceae (15.3%), Rhodobacteraceae (8.3%), Pseudoalteromonadaceae (5.8%), and Vibrionaceae (5.1%) (Fig. 1; Additional file 1: Table S3). Microbiomes of mechanically wounded corals were populated by Proteobacteria and Bacteroidetes (Additional file 1: Tables S4 and S5), while predated corals were mainly dominated by Proteobacteria (Additional file 1: Tables S6 and S7). More specifically, the common coral symbiont Hahellaceae dominated bacterial communities in mechanically wounded corals at Ti (83.9%) and T_f_ (59.9%) (Fig. 2; Additional file 1: Tables S4 and S5). Distinct community assemblages were also observed between parrotfish mouths and predated corals at Ti (Fig. 2; Additional file 1: Tables S1 and S2; pairwise Adonis; *p* = 0.003) and at T_f_ (pairwise Adonis, *p* = 0.012). Among the 66 (Ti) and 49 (T_f_) bacterial families identified in bitten corals at Ti and T_f_, respectively, only a few were represented at relative proportions greater than 1%. These included Hahellaceae (21.6%), Amoebophilaceae (17.5%), and Rivulariaceae (9.7%) (Additional file 1: Table S6) at Ti, and Rhodobacteraceae (13.7%), Pseudoalteromonadaceae (11.0%), Verrucomicrobiaceae (5.9%), Alteromonadaceae (5.5%), Flavobacteriaceae (3.9%), Vibrionaceae (3.9%), Oceanospirillaceae (3.3%), Colwelliaceae (2.5%), Lentisphaeraceae (2.4%), Francisellaceae (1.6%), Paenibacillaceae (1.4%), and Hahellaceae (12.2%) at T_f_ (Additional file 1: Table S7).

#### Field survey

Similar to our mesocosm experiment, microbiomes of parrotfish mouths were dominated by Proteobacteria and Bacteroidetes (Additional file 2: Figure S2) and showed distinct bacterial communities compared to naturally unbitten corals in the field (Additional file 1: Tables S8 and S9; pairwise Adonis; *p* = 0.002). Among the 99 bacterial families identified in fish mouths, only 7 were represented at moderate abundance (1–20%), including Moraxellaceae (16%), Alteromonadaceae (8.5%), Rhodobacteraceae (6.5%), Vibrionaceae (4.6%), Flavobacteriaceae (4.3%), Rhodospirillaceae (4.3%) and Paenibacillaceae (3.3%) (Fig. 2; Additional file 1: Table S10). In naturally unbitten corals, 51 families were identified (Additional file 1: Table S11), and as with the mechanically wounded corals in our mesocosm experiment, microbiomes were mainly populated by Hahellaceae (89.9%) (Additional file 1: Table S11). Differences in bacterial community composition were also observed between parrotfish mouths and naturally bitten corals (Fig. 2; Additional file 1: Tables S8 and S9; pairwise Adonis; *p* = 0.0015. In naturally bitten corals, families with relative abundances exceeding 1% included Hahellaceae (63.4%), Rhodobacteraceae (7.3%), Paenibacillaceae (3.1%), Flavobacteriaceae (1.9%), Rhodospirillaceae (1.6%), Moraxellaceae (1.4%), Alteromonadaceae (1.4%), and Flammeovirgaceae (1.3%) (Additional file 1: Table S12). Finally, we found that bacterial assemblages of water samples significantly differed from parrotfish mouths (Additional file 1: Table S9; pairwise Adonis; *p* = 0.002), as well as unbitten (pairwise Adonis; *p* = 0.003) and bitten corals (pairwise Adonis; *p* = 0.003), suggesting that changes in the coral’s microbial composition are not solely driven by microbial communities in the surrounding reef environment.

### Parrotfish predation increased alpha diversity of *P. lobata* microbiomes

**Fig. 3:**
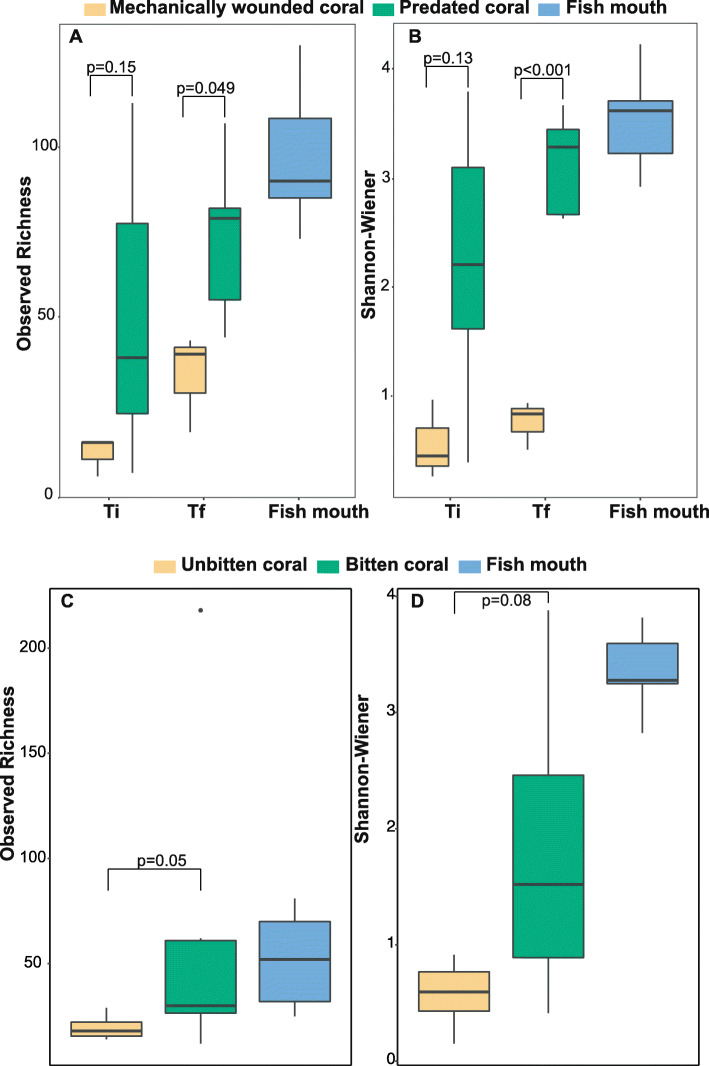
Alpha diversity metrics. Observed richness and Shannon-Wiener diversity indices for the mesocosm experiment (**a**, **b**) and field survey (**c**, **d**) for each sample type and timepoint. *P*-values represent pairwise comparisons, using Tukey’s or Dunn’s test, at each time point between **a**, **b** predated and mechanically wounded corals, **c**, **d** bitten and unbitten corals

Parrotfish predation induced significant changes in the alpha diversity of *P. lobata* microbiomes, both in the mesocosm and in the field. Overall, parrotfish mouth microbiomes showed greater bacterial richness compared to mechanically wounded and predated corals in the mesocosm experiment at both time points (Additional file 1: Tables S13 and S14; pTi = 0.002 and pT_f_ = 0.002) and to unbitten corals in the field (Additional file 1: Tables S15 and S16, *p* = 0.003). However, Shannon-Wiener bacterial diversity did not significantly differ between parrotfish mouths and both predated corals in the mesocosm (Additional file 1: Table S14, pTi = 0.17, pT_f_ = 0.18) and naturally bitten corals in the field (Additional file 1: Table S16, *p* = 0.1). While patterns of alpha diversity in the mesocosm were similar between mechanically wounded and predated corals at Ti (Fig. 3a, b; Additional file 1: Tables S13 and S14; Richness – *p* = 0.15; Shannon – *p* = 0.13), predated corals at T_f_ exhibited 2x greater microbial richness (73.4 ± 11) and 4x greater Shannon-Wiener diversity (3.1 ± 0.2) compared to mechanically wounded corals (Richness: 32.6 ± 8.4 and Shannon: 0.72 ± 0.13) (Fig. 3a, b; Additional file 1: Table S14; Richness – *p* = 0.049; Shannon – *p* < 0.001). In the field, naturally bitten corals exhibited 3x greater microbial richness (62.1 ± 26.9) and diversity (1.8 ± 0.5) compared to unbitten corals, although only differences in richness were significant (Richness: 19.25 ± 1.8 and Shannon: 0.58 ± 0.1) (Fig. 3c, d; Additional file 1: Tables S15 and S16; Richness – *p* = 0.04; Shannon – *p* = 0.08).

### Corallivory generated a distinct microbiome community structure in *P. lobata*

Evidence for changes in microbial community composition following parrotfish predation was present in both our mesocosm experiment and field survey (Fig. 2, Additional file 2: Figures S3 and S4). In the former, predated and mechanically wounded corals exhibited similar patterns in their overall microbial community assemblages at Ti (Additional file 1: Table S2, pairwise Adonis; *p* = 0.07). However, five sub-operational taxonomic units (sOTUs) had greater abundance in predated corals when compared to mechanically wounded corals at Ti. These included members of the Rivulariaceae (genus *Rivularia*; sOTU_15), Phormidiaceae (sOTU_12) and Amoebophilaceae (clade *SGUS912*; sOTU_195) families, as well as two taxa from the orders Nostocales (sOTU_18) and Rhizobiales (sOTU_697) (Additional file 1: Table S17; log2 fold-change 7.05 to 22.9). Among these, three taxa were identified only in predated corals (sOTU_15, sOTU_12, sOTU_697; Additional file 1: Table S18). One sequence (sOTU_195) was found both in mechanically wounded and predated corals (Additional file 1: Table S18). Moreover, the specific sOTU_18 was found both in predated corals and in low abundance (0.12%) in fish mouths, (Additional file 1: Table S18) but not in mechanically wounded corals.

**Fig. 4:**
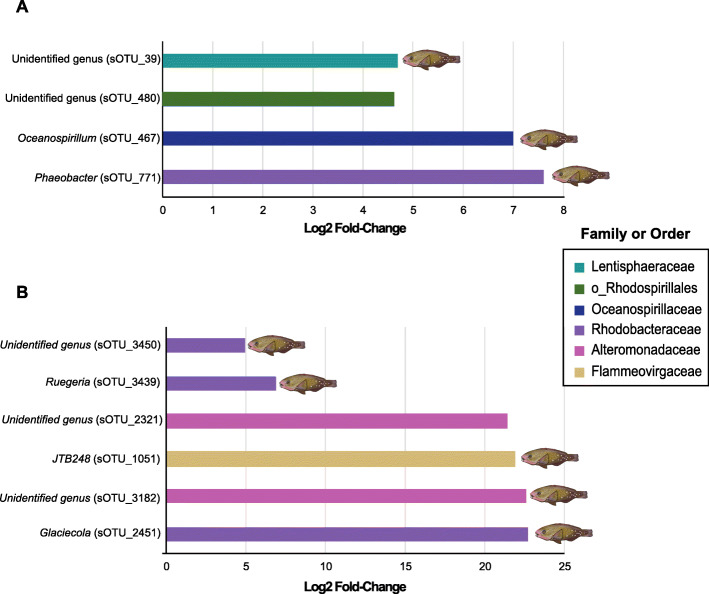
Differential abundance analysis (DESeq2) illustrating the sOTUs labeled as genera and families or order that differed significantly between (**a**) predated and mechanically wounded corals for the mesocosm experiment at T_f_ (48 h) and (**b**) naturally unbitten and bitten corals in the field survey. Fish illustration indicates the presence of the corresponding taxa in parrotfish mouths (image credit: Katrina Munsterman)

At 48 h, mechanically wounded and predated corals exhibited significantly different bacterial community composition (Fig. 2 and Additional file 2: Figure S3; Additional file 1: Table S2; pairwise Adonis; *p* = 0.018). This coincided with a greater abundance of four sOTUs (Fig. 4a; Additional file 1: Table S19; log2 fold-change 4.6 to 7.6), including members of the families Rhodobacteraceae (genus *Phaeobacter*; sOTU_771), Oceanospirillaceae (genus *Oceanospirillum*; sOTU_467), and Lentisphaeraceae (sOTU_39), and the order Rhodospirillales sOTU_480). Of these taxa, two were absent from mechanically wounded corals, but present in relatively low abundance in predated corals (sOTU_771–1.67%; sOTU_467–0.9%), as well as parrotfish mouths (sOTU_771–0.5%; sOTU_467–0.012%) (Additional file 1: Table S18). One taxon (sOTU_480) was found in low abundance in mechanically wounded corals (0.2%) and at moderate levels in predated corals (13%; Additional file 1: Table S18). Finally, sOTU_39 was present at low abundance in mechanically wounded corals (0.04%), but was more abundant in predated corals (2.3%) and parrotfish mouths (2.8%; Additional file 1: Table S18).

**Fig. 5:**
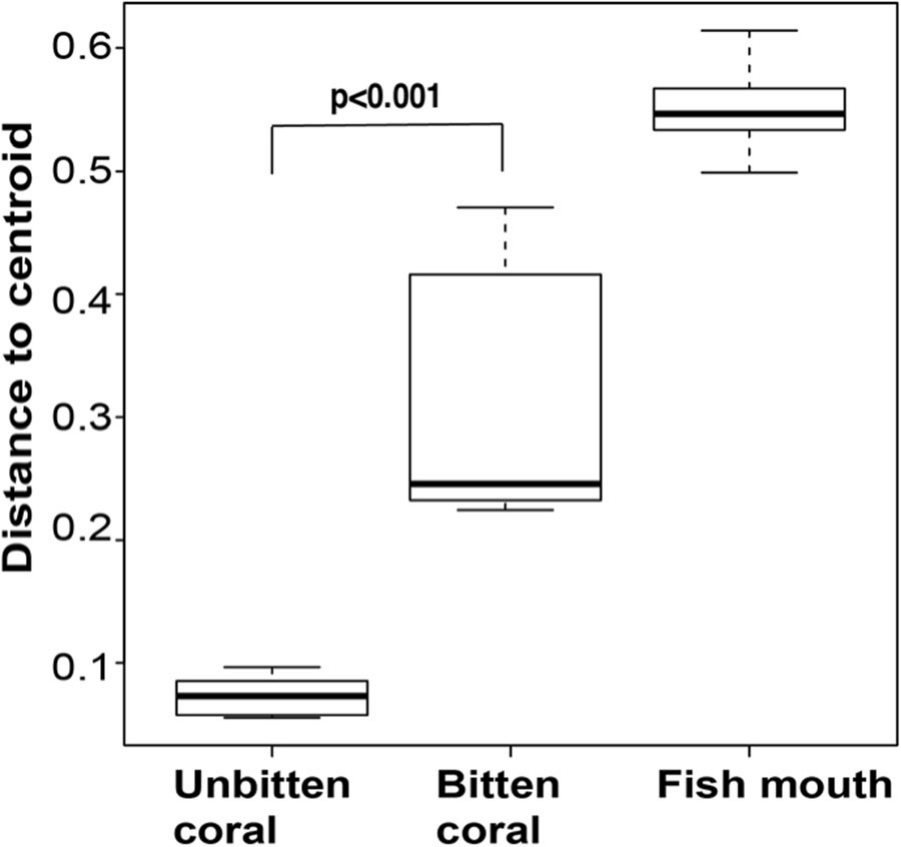
Boxplots illustrating the level of community variability among sample types for the field experiment. *P*-values represent pairwise comparisons, using Tukey’s test, between unbitten and bitten corals

In the field, naturally bitten and unbitten corals showed distinct patterns in bacterial community composition (Fig. 2 and Additional file 2: Figure S4, Additional file 1: Table S9; pairwise Adonis; *p* = 0.01). This coincided with a greater abundance of six sOTUs in naturally bitten compared to unbitten corals (Fig. 4b; Additional file 1: Table S20; log2 fold-change 4.9 to 22.7). Among these, three taxa were undetectable in unbitten corals but present in relatively low abundance in parrotfish mouths, including sequences from the families Flammeovirgaceae (genus JTB248 – sOTU_1051–0.44%; Additional file 1: Table S21), Rhodobacteraceae (genus *Glaciecola* – sOTU_2451–1.7%) and Alteromonadaceae (sOTU_3182–0.27%). Two members from the Rhodobacteraceae family (sOTUs_3439, 3450) were identified in naturally bitten and unbitten corals, as well as in fish mouths (sOTU_3439–1.01%; sOTU_3450–2.41%; Additional file 1: Table S21), while a sequence from the family Alteromonadaceae (sOTU_2321) was found only in naturally bitten corals (0.3%; Additional file 1: Table S21). Finally, we observed 4x greater community variability in the microbiome of naturally bitten corals (0.32 ± 0.04) compared to unbitten corals (0.07 ± 0.005; Fig. 5; Additional file 1: Tables S22 and S23; Tukey HSD - *p* < 0.001).

## Discussion

A number of corallivores are suspected to facilitate the enrichment and/or transmission of microbes within reef-building corals [14, 16], including consumers such as parrotfishes that play key roles in regulating reef ecosystem processes [13]. Using a combination of mesocosm- and field-based approaches, we demonstrated that corallivory by the parrotfish species *Chlorurus spilurus* leads to significant changes in bacterial community composition of *Porites lobata*. In particular, these changes included greater abundances of potential beneficial bacterial taxa and opportunists, some of which were naturally occurring in parrotfish mouths. Our findings indicate that parrotfishes may play an important role in driving the structure of coral microbial communities, either by acting as vectors and/or by facilitating the enrichment of bacteria in reef corals via corallivory.

### Parrotfish-induced *P. lobata* microbiome changes in mesocosm

Patterns of alpha and beta diversity in our mesocosm experiment were similar at T_i_ for mechanically wounded and predated corals. However, five taxa were already observed in greater abundance in predated corals compared to those that were mechanically wounded. All were present at relatively low abundances (< 10%) in predated corals, but may have the potential to affect coral microbiomes and health. For instance, cyanobacteria from the Nostocales order (sOTU_18) are often found in fish guts [25] and were associated with diseased corals [26]. Members of the clade *SGUS912* (sOTU_195) are commonly present in corals exposed to sewage and wastewater outfalls [27]. Taxa from the orders Oscillatoriales (sOTU_12) and Rhizobiales (sOTU_697), and filamentous Cyanobacteria from the genus *Rivularia* (sOTU_15), were associated with stressed and diseased corals and sponges [9, 28–30]. Whether and how these changes affect coral health and fitness, especially when coupled with other stressors, should be investigated further.

At the end of the experiment (T_f_), we observed greater bacterial richness and diversity in predated corals compared to mechanically wounded corals. Patterns of increased alpha diversity are often associated with numerous physical and biotic stressors including water pollution [31, 32], elevated temperature [33, 34], ocean acidification [35], algal competition [36, 37], mechanical wounding, and snail corallivory [38, 39]. However, other studies demonstrated no changes or a significant decrease in microbial diversity and/or richness following mechanical injury [38, 40]. These differences among studies may indicate that responses of coral microbiomes differ due to biological vs. mechanical wounding, or that stressor-induced impacts may be variable depending on coral species or genotypes, local environmental conditions, and/or exposure time. In the present study, increases in bacterial richness and diversity coincided with a compositional shift in bacterial assemblages in predated corals compared to mechanically wounded ones. In addition, microbiomes of predated corals were characterized by moderate abundance (59.9% at T_f_) of the putative beneficial symbiont Hahellaceae at 48 h when compared to Ti (83.9%). Lower abundance of Hahellaceae bacterial taxa is a pattern previously reported in stressed, mechanical injured, and predated corals [9, 38, 39, 41].

In addition, bacterial communities of corals exposed to predation were dominated by members of the families Rhodobacteraceae, Pseudoalteromonadaceae, Alteromonadaceae, Verrucomicrobiaceae and Flavobacteriaceae – taxa that are often associated with both stressed and healthy coral colonies [32, 42], and were also found in relatively high abundance in parrotfish mouths. Four sOTUs were present in greater abundance among predated corals compared to mechanically wounded ones, including taxa from the genera *Phaeobacter* (sOTU_771) and *Oceanospirillum* (sOTU_467), as well as sequences from the Lentisphaerae (sOTU_39) and Rhodospirillales (sOTU_480) orders. Their potential influences on corals may be diverse – ranging from beneficial to opportunistic. Members of the genus *Phaeobacter* were previously found in corals and jellyfish [43–45] and were linked to the production of antibacterial compounds in fishes [46, 47]. Bacteria from the genus *Oceanospirillum* are frequently observed in healthy coral colonies [48, 49], while members of the phylum Lentisphaerae are common in the fish gut [50] and healthy corals [48]. Sequences from the order Rhodospirillales are commonly found in high abundance in stressed and diseased coral colonies [9, 51–53], indicating an opportunistic character. Given that our experiment lasted for 48 h, the persistence of potential beneficial symbionts and opportunistic bacterial taxa and their consequences on coral microbiomes and health will have to be further investigated over longer time period.

### Microbiomes of naturally bitten vs. unbitten *P. lobata* in the field

Microbiomes of *P. lobata* corals found in the field reinforced findings from our mesocosm, as naturally bitten corals exhibited greater bacterial richness compared to unbitten corals. It is worth noting that corals of all treatments, from both the mesocosm experiment and field survey, exhibited relatively low bacterial richness compared to previous work [54]. However, lower richness has consistently been observed among corals inhabiting reefs in Mo’orea [39, 55] and may be related to greater community dominance by members of the Hahellaceae family.

In our study, increased bacterial richness in naturally bitten was associated with a shift in bacterial community composition compared to unbitten corals. Bitten corals were mainly populated by potential opportunistic bacterial taxa, including sequences from the families Rhodobacteraceae, Paenibacillaceae, Flavobacteriaceae, Rhodospirillaceae, Moraxellaceae, Alteromonadaceae, and Flammeovirgaceae [9, 56–58], all of which are taxa that were also present in relatively high abundance in parrotfish mouths. Significant changes in community composition were associated with greater abundance of six taxa in naturally bitten vs. unbitten corals. Among them, three represented the Flammeovirgaceae (genus *JTB248;* sOTU_1051), and Alteromonadaceae (sOTU_2321; sOTU_3182;) families, that were previously associated with stressed, aged, and/or diseased corals [59–61]. Three other taxa were assigned to the Rhodobacteraceae family, taxa commonly associated with both healthy and stressed corals [56, 62]. In particular, a strain from the genus *Ruegeria* was found in lesioned and diseased corals [41, 56] and is known to inhibit growth of the coral pathogen *Vibrio coralliilyticus* [63].

As with our mesocosm experiment, our field survey identified taxa with potential beneficial and/or deleterious implications for coral microbiomes, health, and fitness. Further studies are needed to understand the functional roles of these microbes and their interplay with coral stressors. Finally, we observed greater bacterial compositional variability in naturally bitten compared to unbitten corals. Increased microbiome variability is consistent with previous studies showing that numerous animals, including corals, exhibit elevated community variability when exposed to stressors (i.e. the Anna Karenina Principle [64];), such as mechanical wounding [39]. This further indicates the potential for parrotfish to increase dysbiosis susceptibility in corals via corallivory*.*

### Potential parrotfish-mediated bacterial transmission and enrichment pathways in *P. lobata*

Parrotfish predation could alter the microbiomes of *P. lobata* via several pathways, including i) direct transmission of bacteria from fish mouths to the coral mucus/tissue layer, ii) indirectly facilitating bacterial invasion from the surrounding environment following wounding, iii) indirectly facilitating growth of bacterial taxa already present within the coral microbiomes or from the surrounding environment, and iv) a combination of these three pathways. We observed evidence for each of these possible pathways in our experiments. For example, evidence that parrotfish may directly transmit bacteria to *P. lobata* was observed both in the mesocosm experiment and field survey. In the former case, a taxon from the order Nostocales (sOTU_18) at Ti, as well as two taxa from the genera *Phaeobacter* (sOTU_771) and *Oceanospirillum* (sOTU_467) at T_f_, were both found in predated corals and fish mouths, but not in mechanically wounded corals. This indicates that mechanical wounding was insufficient to introduce these taxa and that they were likely vectored via parrotfish predation. Similar patterns were observed in the field, with sequences from the families Flammeovirgaceae (sOTU_1051; genus *JTB248*), Rhodobacteraceae (sOTU_2451; genus *Glaciecola*), and Alteromonadaceae (sOTU_3182) present only in bitten corals and fish mouths – not unbitten corals. We also observed evidence that predation may facilitate the invasion of bacterial taxa from the surrounding environment. At T_i_ in our mesocosm experiment, three potential opportunistic bacterial coral taxa were found in predated corals, but not in mechanically wounded corals or parrotfish mouths (sOTU_12, sOTU_15 and sOTU_697). Similarly, in the field, sequences from the family Alteromonadaceae (sOTU_2321) were only found in naturally bitten corals, indicating enrichment from the surrounding environment.

We also observed potential enrichment from microbes preexisting on corals and/or from the external environment, such as members from the clade *SGUS912* (sOTU_195) and the Rhodospirillales order (sOTU_480), which were identified in predated and mechanically wounded corals – but not fish mouths – at T_i_ and T_f_, respectively. Finally, evidence from both experiments suggested a combination of different pathways including transmission and/or enrichment. In the manipulative experiment, taxon sOTU_39 from the Lentisphaerae order was present in moderate abundance in predated corals, as well as in low abundance in mechanically wounded corals and parrotfish mouths at T_f_. In the field, two taxa from the Rhodobacteraceae family (sOTU_3439, sOTU_3450) were present in fish mouths and bitten corals, as well as in relatively low abundance in unbitten corals.

Collectively, our findings suggest that parrotfish corallivory may be an important driver structuring coral-associated bacterial communities. Evidence that parrotfish vector and/or facilitate the enrichment of bacteria within corals, both in our mesocosm experiment and field surveys, was surprisingly consistent – especially given that sampling of corals and parrotfish mouths was conducted haphazardly in the back reef during our field surveys. This suggests that parrotfish mouths may harbor a consistent microbial signature in the studied reef area that allows *C. spilurus* to vector rare taxa via corallivory. Our findings add to growing body of evidence demonstrating the potential for corallivores, such as snails (*Drupella spp*., *Coralliophila spp.*), crown-of-thorn sea stars (*Acanthaster spp.*) and worms (*Hermodice caniculata*), to vector and/or facilitate the enrichment of microbes in corals [38, 65, 66]. Our study is the first to document such potential in parrotfishes, adding to their key roles as corallivores, bioeroders, and herbivores on coral reefs. Previous work suggests that other candidate species, such as butterflyfishes, are unlikely to vector microbes [16, 67] – potentially due to their distinct “browser” feeding mode (but see [17]). In contrast, the “scraper” and “excavator” feeding modes of many parrotfishes may make them ideal candidates to transmit microbes to corals. The interplay between these abilities and the other critical roles of parrotfishes on coral reefs will be of considerable interest for reefs of the future.

## Conclusion

Our findings provide evidence that parrotfish corallivory can have important effects on coral microbiomes, with the potential to impact coral health. *C. spilurus* predation both in the laboratory and field induced an increase in alpha diversity and a compositional shift in the microbial assemblages of *P. lobata* corals, which coincided with a greater abundance of potential beneficial bacteria (i.e. *Ruegeria*, *Phaeobacter*) as well as opportunistic taxa (i.e Flammeovirgaceae, Rhodospirillaleceae, *Glaciecola).* Importantly, several taxa were undetectable on mechanically wounded and naturally unbitten corals but present in predated, naturally bitten corals and in parrotfish mouths, suggesting parrotfish vector new bacteria to corals during predation. However, the ability of *C. spilurus* to vector and/or facilitate the enrichment of microbial opportunists, as well as increase microbiome variability, in naturally bitten *P. lobata* corals is consistent with recent findings linking nutrient pollution and parrotfish predation to coral mortality [13]. This suggests that common trophic interactions may increase coral susceptibility to dysbiosis, especially when corals are already stressed from other factors such as nutrient pollution, temperature, or sedimentation. Together, our results shed light on underappreciated pathways linking parrotfishes to microbial enrichment and dysbiosis within reef corals. Future work should investigate the interactive effects of parrotfish corallivory and abiotic stressors (e.g. nutrient pollution and ocean warming) to evaluate their consequences for coral microbiomes and fitness.

## Material and methods

### Assessing *Chlorurus spilurus* diet

We quantified feeding behavior of *C. spilurus* in situ to examine how frequently this species preys on live coral. A diver conducted 20-min timed follows at two backreef locations in the lagoon of Mo’orea (French Polynesia) during the months of July – August in 2017 and 2018. We followed 23 focal individuals during peak grazing hours to control for temporal variation in foraging behavior (1000–1600 [68];). Targeted substrates were binned into bites either on live and dead corals, including rubble and pavement (turf algae, farmers turf and crustose coralline algae). We focused on *C. spilurus* > 150 mm as these individuals are most likely to bite live corals.

### Sample collections and experimental design

Experiments were performed in July 2017 in Mo’orea, French Polynesia at the University of California Gump Research Station. For the mesocosm experiment, we collected 7 individual bullethead parrotfish (*Chlorurus spilurus*; Fig. 1a) (~ 200 mm total length) at ~ 3 m depth, in the back reef area along the north shore of Mo’orea (17°28′50.6″S 149°48′59.4″W) using hand and barrier nets. We also collected 10 apparently healthy *Porites lobata* colonies (~ 20 cm diameter) at the same depth and location. Fish and corals were immediately transferred to the Gump Research Marine Station. Once at the research station, parrotfish and coral colonies were placed into two independent mesocosms of 1155 L volume (Pentair AES Polyethylene Tank) that were supplied with flow-through seawater originating from the reef adjacent to the station. Shade cloth was applied uniformly on each mesocosm to moderate light intensity and promote acclimation of corals and fish. The day following collections, we sampled microbes from the mouth of each *C. spilurus* individual by carefully swabbing the inner side of the beak with sterile culture swabs (BD CultureSwab, BD). After sampling the mouth microbiome of all 7 fish, each fish was assigned to a single *P. lobata* colony and was coaxed into biting the colony two times at separate locations to create two independent feeding wounds. For microbial analyses of predated coral colonies, a sample of coral tissue was collected from one of the two wounds immediately after parrotfish had bitten the colony (Ti). Each colony’s other bite wound was sampled 48 h later (T_f_). Samples were collected using a sterile bone cutter to remove a portion of the coral tissue (tissue, mucus and a small part of the skeleton) approximately 1 × 1 cm at the bite location. Rather than investigating how artificial wounding would affect coral microbiomes [39], we were specifically interested in understanding how parrotfish corallivory changed coral microbiomes. Therefore, instead of comparing the microbiome of corals with parrotfish predation to corals without parrotfish bites, we used mechanically wounded corals as controls. To compare how parrotfish vs. mechanical wounds affected coral microbiomes, the 3 remaining colonies of *P. lobata* (hereafter “mechanically wounded”) were wounded in two separate locations using sterile bone cutters to mimic the wounds caused by parrotfish. These mechanically wounded corals were then placed in a separate tank and assessed in the same manner as described above. The resulting lesions from both parrotfish and artificial wounding were ~ 2 mm deep and 1 cm long and removed coral tissue as well as part of the skeleton. Due to logistical limitations at the station, all of the parrotfish wounded corals were placed in one mesocosm, while all of the mechanically wounded corals were placed in a second mesocosm. Both mesocosms were fed flow though seawater via a common seawater source system and were physically adjacent to each other assuring similar light levels.

For our field study, we selected a 500 m stretch of shallow back reef area on the north shore of Mo’orea (17°28′35.2″S 149°47′34.9″W). Ten *C. spilurus* individuals (~ 20 cm total length) were collected using the same methods as described above. On the boat, individual parrotfish were transferred into large coolers equipped with bubblers and filled with seawater from the reef area. Microbial samples from each parrotfish mouth were directly collected using sterile swabs (BD CultureSwab, BD), as described above. Parrotfish were then released back onto the reef. The same day, we haphazardly selected *Porites lobata* colonies (20 cm diameter) in the back reef area with significant evidence of recent predation by parrotfishes (hereafter “bitten”), as well as colonies with no evidence of predation (hereafter “unbitten”; *n* = 10 colonies per wound status; Fig. 1b, c). We sampled a segment of coral tissue (1 × 1 cm) from the surface of each colony in situ using a sterile bone cutter. Bitten corals were sampled at a bite location chosen haphazardly on the colony, while unbitten corals were sampled at a haphazard location on the colony. Tissue collection was performed in situ across the designated reef an approximately 500 m stretch of lagoon. One liter water samples (*n* = 4) were collected haphazardly across the reef and filtered on a 0.2 μm Millipore filter. Sterile swabs and both coral and water samples were placed on ice in coolers until reaching the station where they were frozen at − 80 °C prior to microbial analyses.

### DNA extraction and 16S rRNA gene amplification

High-throughput sequencing of the 16S rRNA gene was used to compare microbiome diversity, composition, and stability metrics. DNA extraction was performed using the DNeasy PowerSoil Kit (Qiagen) according to manufacturer instructions. AccuStart II PCR ToughMix (Quanta BioSciences, Gaithersburg, Maryland, USA) was used to perform two-step Polymerase Chain Reaction (PCR) on the V4 hypervariable region of the 16S rRNA gene using the primer pair 515FY (5′-GTGYCAGCMGCCGCGGTAA-3′) [69] and 806RB (5′-GGACTACNVGGGTWTCTAAT-3′) [70] targeting bacterial and archaeal communities. For each reaction, 6.25 μl AccuStart II ToughMix (2X), 1.25 μl forward primer (10 μM), 1.25 μl reverse primer (10 μM), 0.5 μl sample DNA, and 3.25 μl PCR-grade water was used. PCR amplification consisted of a 3 min denaturation at 94 °C followed by 35 cycles of 45 s at 94 °C, 60 s at 50 °C, and 90 s at 72 °C, and ending with 10 min extension step at 72 °C. A 1.5% agarose gel was run with amplified products which were manually excised to purify the 16S target band using Wizard® SV Gel and PCR Clean-Up System (Promega). The resulting products were then custom barcoded in a second PCR reaction with 12.5 μl ToughMix (2X), 9.5 μl water, and 1 μl of gel-purified sample DNA. The 12-cycle barcoding reaction consisted of a 5 min denaturation at 95 °C, 30 s melting at 95 °C, 3 min annealing at 63 °C, 30 s extension at 72 °C, ending with a 10 min hold at 72 °C. Barcoded amplicons were pooled in equivolume ratios and purified using Agencourt® AMPure XP beads. Prepared library pools were sequenced at the Center for Genome Research and Biocomputing (CGRB) at Oregon State University (OSU) on the Illumina MiSeq platform using MiSeq reagent kit v.3 (2 × 300 bp paired-end reads).

### Data processing of mesocosm experiment samples

In association with the mesocosm experiment, a total of 50 samples were run through the data processing pipeline, 7 fish samples, 40 coral samples, 2 negative samples, and a positive control. Using VSEARCH v2.8.1 [71], sequences were truncated at the first position having a quality score less than or equal to 10, paired-end reads were merged, and merged reads with a total expected error > 1 per base or with > 1 N were discarded. This resulted in a total of 502,502 reads. Next, the Deblur workflow was used to trim quality-controlled reads to 250 base pairs, to identify exact sequences with single-nucleotide resolution, and to filter de novo chimeras [22]. This process resulted in 42 samples with 179,293 reads after 8 samples were lost in the Deblur workflow.

Next, the QIIME2 pipeline (https://qiime2.org [72];) was then used to process the OTU table resulting from the Deblur workflow. Taxonomy was assigned against the GreenGenes database [73], which is commonly used in microbial analyses [74], using classify-sklearn algorithm in QIIME2. Unassigned OTUs, singletons, and mitochondria or chloroplast sequences were removed from the OTU table. This removed a total of 7149 reads from the dataset. The number of sequences per sample type following filtering varied from 829 to 10,284 for coral tissue and from 3440 to 14,020 for fish mouthpart samples. Samples were then rarefied to a depth of 829 reads which resulted in the loss of 12 samples with insufficient read depth. The pre-filtered unprocessed sOTU table, metadata and associated negative control taxonomy table can be found in the Additional file 1: Tables S24 and S25. Rarefaction was performed using the function rarefy_even depth in the package phyloseq (v.1.26.1) in R. Four more samples were removed from the dataset as they were collected during a sampling timepoint that was ultimately excluded from these analyses due to low replication following the processing described above.

### Data processing of field survey

A total of 139 samples were collected in the field and run through the data processing pipeline, including coral tissue, fish mouthparts, fish feces, sediment, water, and negative controls. Only a subset of these samples, 34, were relevant to the questions of this study and included in the analyses.

The following steps represent read counts for the 34 samples included in this analysis. Raw sequences were first demultiplexed then trimmed of primers and adapters resulting in 1,323,828 reads across the 34 samples. Using VSEARCH v2.8.1 [71], sequences were truncated at the first position having a quality score less than or equal to 10, paired-end reads were merged, and merged reads with a total expected error > 1 per base or with > 1 N were discarded. This resulted in a total of 526,544 reads. Next, the Deblur workflow was used to trim quality-controlled reads to 250 base pairs, to identify exact sequences with single-nucleotide resolution, and to filter de novo chimeras [22]. This process resulted in 33 samples with 164,793 after one sample was lost in the Deblur workflow.

Next, the QIIME2 pipeline (https://qiime2.org [72];) was then used to process the OTU table resulting from the Deblur workflow. Taxonomy was assigned against the GreenGenes database [73] using classify-sklearn algorithm in QIIME2. Unassigned OTUs, singletons, and mitochondria or chloroplast sequences were removed from the OTU table. This removed a total of 10,257 reads from the dataset. The number of sequences per sample type following filtering varied from 1551 to 7050 for coral tissue and from 2319 to 10,360 for fish mouthpart samples and 6021 to 8890 reads for water samples. Samples were then rarefied to a depth of 1551 reads which resulting in the loss of 4 samples with insufficient read depth. The pre-filtered unprocessed sub-operational taxonomic unit (sOTU) table, metadata and associated negative control taxonomy table can be found in the Additional file 1: Tables S26 and S27. Rarefaction was performed using the function rarefy_even depth in the package phyloseq (v.1.26.1) in R.

### Data analyses and statistics

Following rarefaction, two alpha diversity metrics were computed – the observed species richness and the Shannon-Wiener index. The effects of sample type for i) mesocosm experiment (mechanically wounded, predated coral, fish mouth) within time periods and ii) the field (naturally unbitten, bitten coral, and fish mouth) experiment on diversity metrics were assessed using analysis of variance (ANOVA) with the function aov in the R package stats (v.3.5.3). When significant, pairwise comparisons among groups were performed using Tukey’s Honest significant differences (Tukey HSD). The assumptions of normality and homoscedasticity of residuals were tested using Shapiro-Wilk and Levene tests, respectively. If not fulfilled, nonparametric tests were performed using Kruskal-Wallis and Dunn’s tests using the function dunnTest within the R package FSA (v.0.8.22).

To illustrate the average relative abundance of the 25 most abundant taxa represented in each sample type at T_f_ (48 h) for both the manipulative and field experiments, we drew two heatmaps and at the family level by agglomerating the 25 taxa using the function tax_glom (including the command NArm = F) within the R package phyloseq (v1.26.1).

Furthermore, to display changes in microbial community composition among samples in either the mesocosm at T_f_ or the field experiments, two distinct non-metric multidimensional scaling (NMDS) on the Bray-Curtis dissimilarity matrices [75] were performed using the function metaMDS in the R package vegan (v.2.5–4). To test for differences in beta diversity among sample types for the manipulative and field experiments, we computed two permutational analysis of variance (PERMANOVA) based on the Bray-Curtis dissimilarity matrices and 999 permutations using the function Adonis in the R package vegan [76]. Subsequent pairwise differences were tested using the function pairwise.adonis in the R package vegan [76]. *P*-values were adjusted according to the false discovery rate, accounting for multiple comparisons.

We used the R package DESeq2 (v1.22.2) [77] on a pre-filtered unrarefied sOTU table to identify which sOTUs exhibited significant abundance among sample types both in the mesocosm experiment (at T_i_ and T_f_) and in the field survey. From the pre-filtered unrarefied sOTU table, we used the function tax_glom within the phyloseq package (v1.26.1) to agglomerate taxa at the genus level (including the parameter NArm = F). DESeq2 incorporates a model based on the negative binomial distribution and includes a Wald posthoc test. *P*-values were adjusted for multiple comparisons using the Benjamini-Hochberg method [78].

Finally, to assess the variability of microbial composition between sampletype we computed an analysis of multivariate homogeneity of group dispersions [79]. This analysis tested whether community variability among samples, measured based on the Bray-Curtis dissimilarity metric, significantly differed between the three sample types, and was performed using the function betadisper in the R package vegan. Due to sample size limitation in the mesocosm experiment, the test was computed on the field survey coral samples only. When community variability significantly differed across sample type, pairwise tests were performed between groups using Tukey HSD. For clarity, findings presented in the result section are described as mean ± SE and *p*-values were considered significant for *p* < 0.05.

## Supplementary information

**Additional file 1: Table S1.** Results of the permutational ANOVA on the bacterial assemblages according to the sample type (control, predated coral and fish mouth) assessed at Ti and Tf for the mesocosm experiment. **Table S2.** Results of pair-wise tests on the effect of the sample type on the bacterial assemblages for the mesocosm experiment at Ti and Tf. **Table S3.** Average relative abundance of the families present in the fish mouths for the mesocosm experiment. **Table S4.** Average relative abundance of the families present in mechanically wounded corals at Ti for the mesocosm experiment. **Table S5.** Average relative abundance of the families present in mechanically wounded corals at Tf. **Table S6.** Average relative abundance of the families present in the predated corals at Ti for the mesocosm experiment. **Table S7.** Average relative abundance of families present in predated corals at Tf. **Table S8.** Results of the permutational ANOVA on the bacterial assemblages according to the sample type assessed for field experiment including or not water samples. **Table S9.** Results of pair-wise tests on the effect of the sample type on the bacterial assemblages for the field experiment. **Table S10.** Average relative abundance of the families present in the fish mouths for the field experiment. **Table S11.** Average relative abundance of the families present in naturally unbitten corals in the field. **Table S12.** Average relative abundance of families present in bitten corals for the field experiment. **Table S13.** Results of ANOVA and non-parametric tests of the effect of the sample type on alpha diversity metrics (Observed Richness and Shannon-Wiener Index) for the mesocosm experiment at Ti and Tf. **Table S14.** Results of posthoc tests assessing the effect of the sample type on alpha diversity metrics (Observed Richness and Shannon-Wiener index) for the mesocosm experiment at Ti and Tf. **Table S15.** Results of ANOVA and non-parametric tests on the effect of the type of sample on alpha diversity metrics (Observed Richness and Shannon-Wiener index) for the field experiment. **Table S16.** Results of posthoc tests on the effect of the sample type on alpha diversity metrics (Observed Richness and Shannon-Wiener index) for the field experiment. **Table S17.** Results from differential abundance analyses (DESeq2) on the effect of the sample type at Ti for the mesocosm experiment. **Table S18.** Average relative abundance of taxa present in greater differential abundance in predated corals compared to mechanically wounded corals for the mesocosm experiment at Ti and Tf. **Table S19.** Results from differential abundance analyses (DESeq2) on the effect of the sample type at Tf for the mesocosm experiment. **Table S20.** Differential abundance analysis for the field experiment according to the sampletype. **Table S21.** Average relative abundance of taxa present in greater differential abundance in naturally bitten corals compared to controls for the field experiment. **Table S22.** Results of Permutation test for homogeneity of multivariate dispersions (betadisper) on the effect of the sample type in the field survey. **Table S23.** Results of Permutation test for homogeneity of multivariate dispersion (betadisper) on the effect of the sample type in the field survey. **Table S24.** filtered unprocessed sOTU table for the mesocosm experiment. **Table S25.** Taxa table for the negative control of the mesocosm experiment. **Table S26.** filtered unprocessed sOTU table for the field survey. **Table S27.** Taxa table for the negative control in the field survey.

**Additional file 2: Figure S1.** Relative abundance (Phyla) of taxa present in mechanically wounded, predated corals and fish mouths for the manipulative experiment at T48h. **Figure S2.** Relative abundance (Phyla) of taxa present in unbitten, bitten corals and fish mouths in the field. **Figure S3.** NMDS displaying the microbial assemblages according to the sample type at T48h for the mesocosm experiment. **Figure S4.** NMDS displaying the microbial assemblages according to the sample type for the field experiment.

## Data Availability

Raw sequences reads associated to these datasets have been deposited in NCBI Short Read Archive (SRA) under the bioproject PRJNA573999. The source code associated to this study is hosted in the Git repository: https://github.com/laylaeb/parrotfish.
