# Supplementary material for: Parrotfish predation drives distinct microbial communities in reef-building corals
Source: Anim Microbiome. 2020 Feb 10;2:5. doi: 10.1186/s42523-020-0024-0 (PMC7807759; doi:10.1186/s42523-020-0024-0)
Supplement: Supplementary file 2 — Additional file 2: Figure S1. Relative abundance (Phyla) of taxa present in mechanically wounded, predated corals and fish mouths for the manipulative experiment at T48h. Figure S2. Relative abundance (Phyla) of taxa present in unbitten, bitten corals and fish mouths in the field. Figure S3. NMDS displaying the microbial assemblages according to the sample type at T48h for the mesocosm experiment. Figure S4. NMDS displaying the microbial assemblages according to the sample type for the field experiment. [file 42523_2020_24_MOESM2_ESM.docx]

*The Following Supplementary accompanies the manuscript*

**Parrotfish predation drives distinct microbial communities in reef-building corals**

*Leïla Ezzat^1*^, Thomas Lamy^1^, Rebecca L. Maher^2^, Katrina S. Munsterman^1^, Kaitlyn M. Landfield^1^, Emily R. Schmeltzer^2^, Cody S. Clements^3^, Rebecca Vega Thurber^2^ and Deron E. Burkepile^1,4^*

^1^Department of Ecology, Evolution and Marine Biology, University of California Santa Barbara, Santa Barbara, CA 93106, USA

^2^Department of Microbiology, Oregon State University, Corvallis, OR, USA

^3^School of Biological Sciences and Aquatic Chemical Ecology Center, Georgia Institute of Technology, Atlanta, GA, USA

^4^Marine Science Institute, University of California Santa Barbara, Santa Barbara, CA, USA

***corresponding author: [leila.ezzat@gmail.com](mailto:leila.ezzat@gmail.com)

**Figure S1**. Relative abundance (Phyla) of taxa present in mechanically wounded, predated corals and fish mouths for the manipulative experiment at T48h.

**
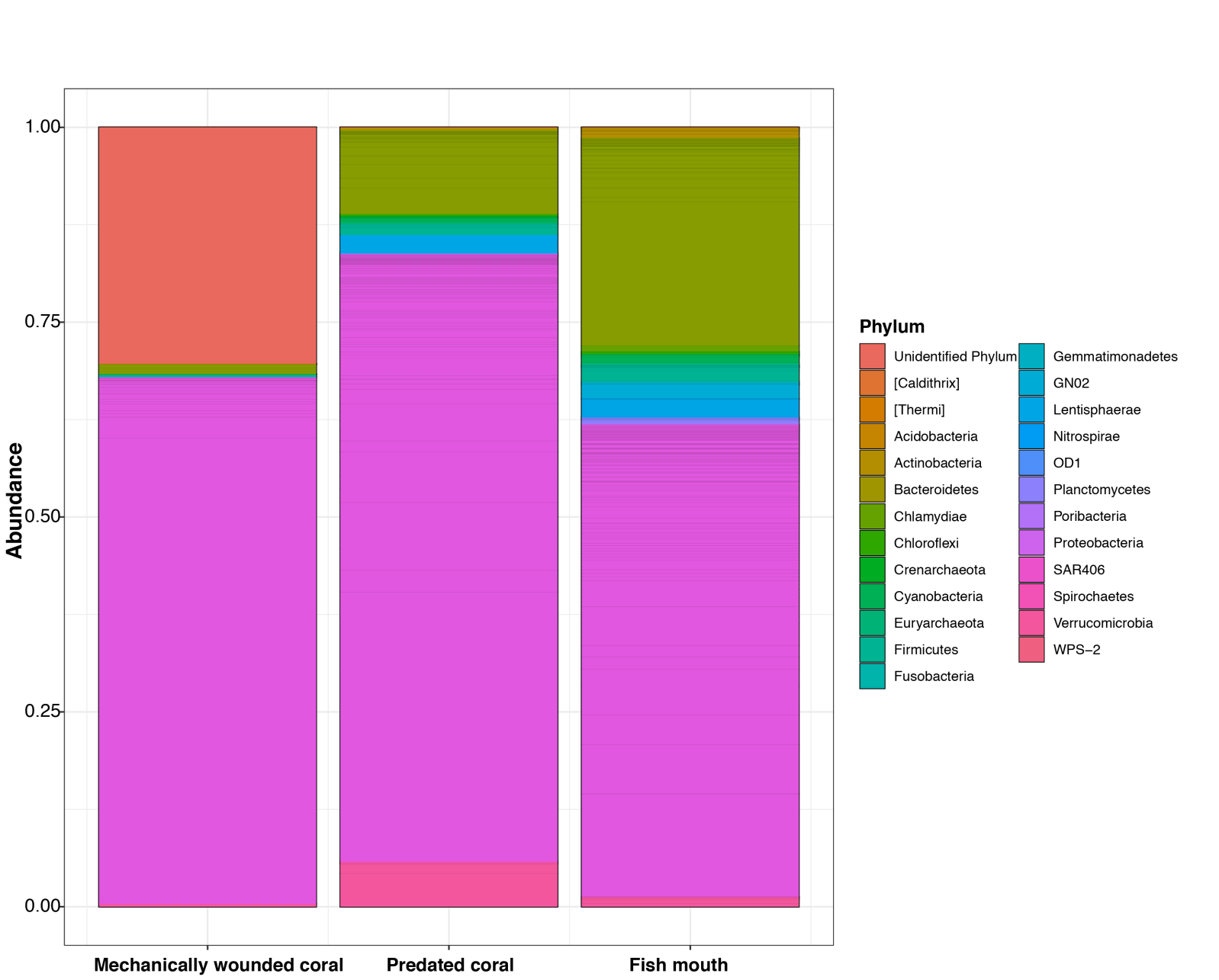
**

**Figure S2.** Relative abundance (Phyla) of taxa present in unbitten, bitten corals and fish mouths in the field.

**
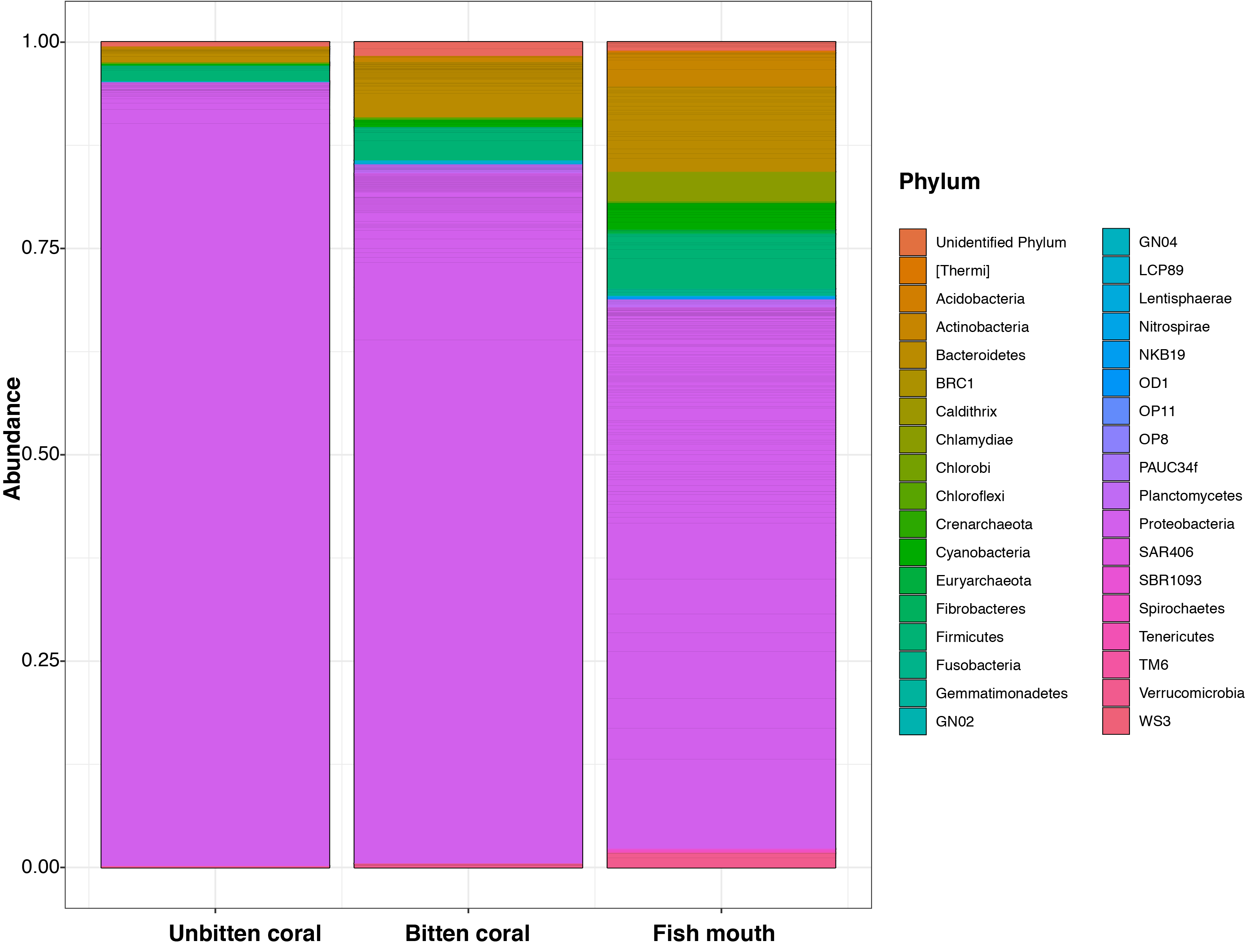
­­**

**Figure S3.** NMDS displaying the microbial assemblages according to the sample type at T48h for the mesocosm experiment

**
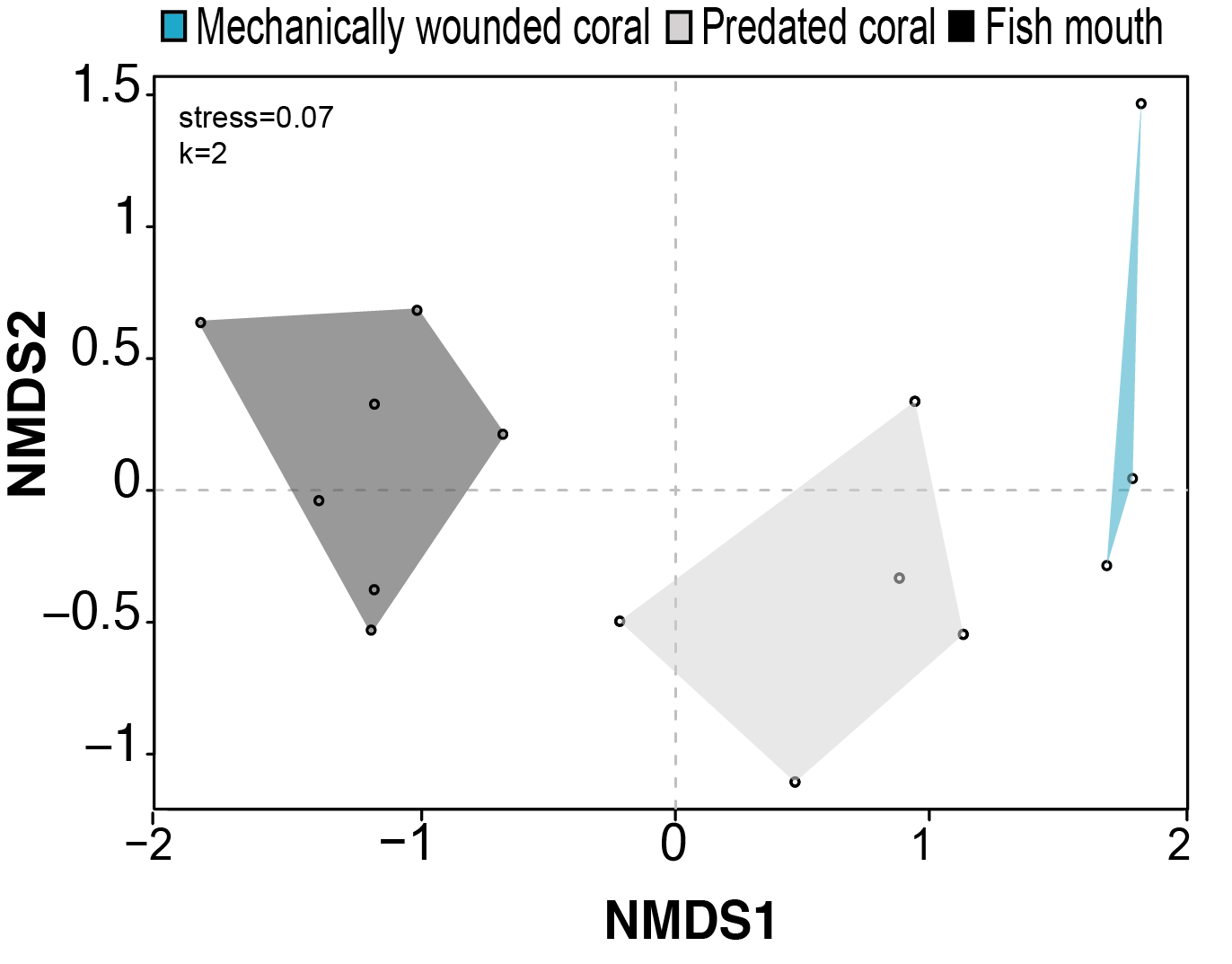
**

**­­**

**Figure S4.** NMDS displaying the microbial assemblages according to the sample type for the field experiment.

**
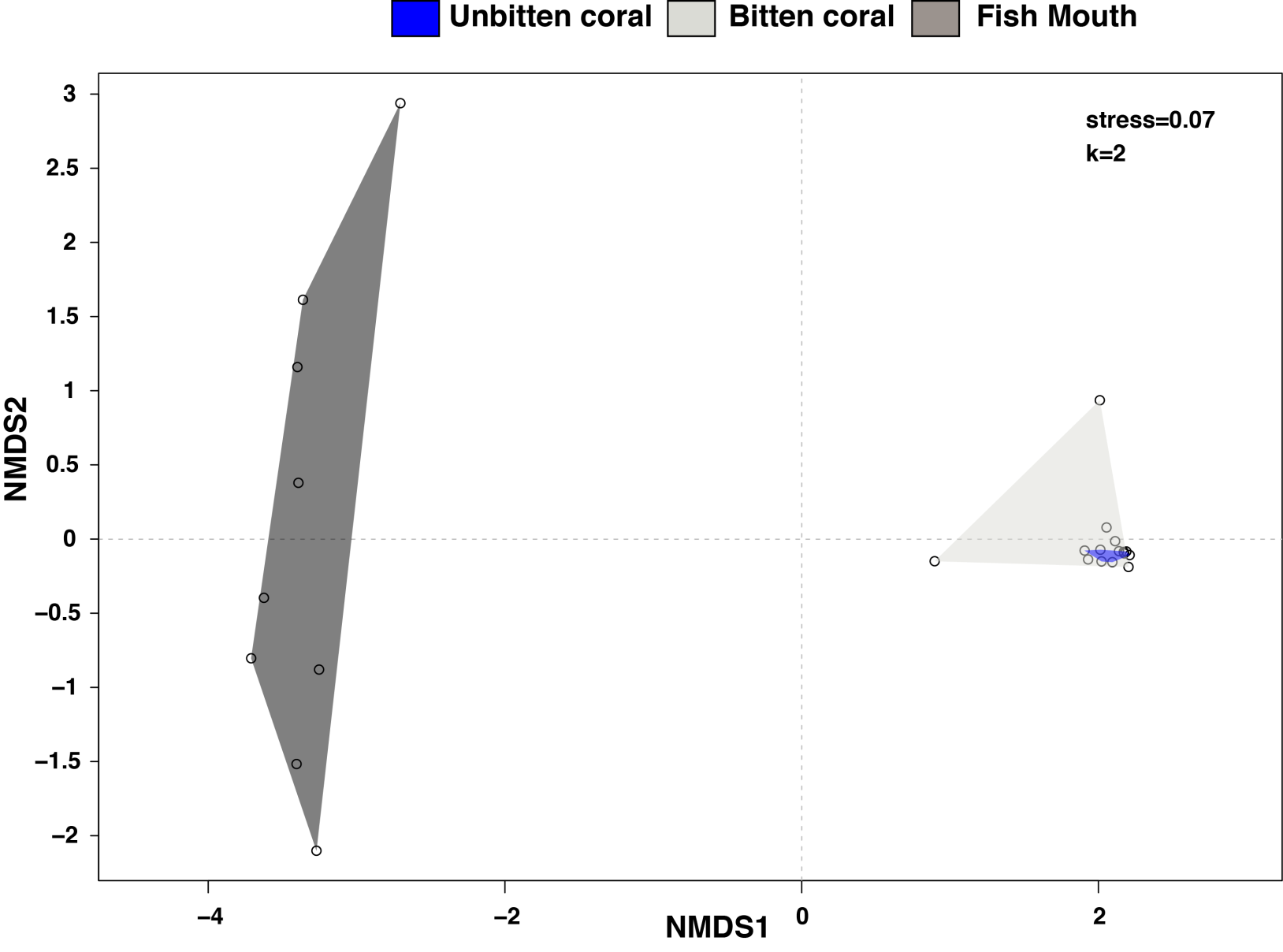
**
